# Cefotaxime Exposure Selects Mutations within the CA-Domain of *envZ* Which Promote Antibiotic Resistance but Repress Biofilm Formation in Salmonella

**DOI:** 10.1128/spectrum.02145-21

**Published:** 2022-04-27

**Authors:** Eleftheria Trampari, Chuanzhen Zhang, Kathryn Gotts, George M. Savva, Vassiliy N. Bavro, Mark Webber

**Affiliations:** a Quadram Institute Bioscience, Norwich, United Kingdom; b Medical School, University of East Anglia, Norfolk, United Kingdom; c School of Biological Sciences, University of Essex, Colchester, United Kingdom; d National Risk Assessment Laboratory for Antimicrobial Resistance of Animal Original Bacteria, College of Veterinary Medicine, South China Agricultural University, Guangzhou, China; e Guangdong Key Laboratory for Veterinary Drug Development and Safety evaluation, College of Veterinary Medicine, South China Agricultural University, Guangzhou, China; University of Pittsburgh

**Keywords:** antimicrobial resistance, biofilms, evolution, two component systems

## Abstract

Cephalosporins are important beta lactam antibiotics, but resistance can be mediated by various mechanisms including production of beta lactamase enzymes, changes in membrane permeability or active efflux. We used an evolution model to study how Salmonella adapts to subinhibitory concentrations of cefotaxime in planktonic and biofilm conditions and characterized the mechanisms underpinning this adaptation. We found that Salmonella rapidly adapts to subinhibitory concentrations of cefotaxime via selection of multiple mutations within the CA-domain region of EnvZ. We showed that changes in this domain affect the ATPase activity of the enzyme and in turn impact OmpC, OmpF porin expression and hence membrane permeability leading to increased tolerance to cefotaxime and low-level resistance to different classes of antibiotics. Adaptation to cefotaxime through EnvZ also resulted in a significant cost to biofilm formation due to downregulation of curli. We assessed the role of the mutations identified on the activity of EnvZ by genetic characterization, biochemistry and *in silico* analysis and confirmed that they are responsible for the observed phenotypes. We observed that sublethal cefotaxime exposure selected for heterogeneity in populations with only a subpopulation carrying mutations within EnvZ and being resistant to cefotaxime. Population structure and composition dynamically changed depending on the presence of the selection pressure, once selected, resistant subpopulations were maintained even in extended passage without drug.

**IMPORTANCE** Understanding mechanisms of antibiotic resistance is crucial to guide how best to use antibiotics to minimize emergence of resistance. We used a laboratory evolution system to study how Salmonella responds to cefotaxime in both planktonic and biofilm conditions. In both contexts, we observed rapid selection of mutants within a single hot spot within envZ. The mutations selected altered EnvZ which in turn triggers changes in porin production at the outer membrane. Emergence of mutations within this region was repeatedly observed in parallel lineages in different conditions. We used a combination of genetics, biochemistry, phenotyping and structural analysis to understand the mechanisms. This data show that the changes we observe provide resistance to cefotaxime but come at a cost to biofilm formation and the fitness of mutants changes greatly depending on the presence or absence of a selective drug. Studying how resistance emerges can inform selective outcomes in the real world.

## INTRODUCTION

Salmonella is one of the most common causative agents of foodborne infections around the world responsible for considerable morbidity and mortality and Salmonella contamination is of particular concern to the food industry (1). It has been estimated that 94 million cases per year are due to nontyphoidal salmonellosis, out of which 80 million cases were associated with contaminated food (2). Most infections by nontyphoidal Salmonella are self-limiting and don’t require antimicrobial treatment. However, immunocompromised individuals, infants and the elderly are at risk of serious infection including invasion of Salmonella to the bloodstream. In these cases, use of antimicrobials is crucial for treatment.

Extended-spectrum cephalosporins, a class of β-lactams, are widely used for the treatment of nontyphoidal Salmonella infections (3–5). They enter the Gram-negative bacterial cell through porins in the outer membrane and block peptidoglycan synthesis, resulting in lysis and cell death (6). Extended use of this class of antibiotics has led to emergence of resistance, which although low in Salmonella is rising, and now over 25% of Salmonella isolates from humans across the EU demonstrate a multidrug resistance phenotype (7).

Common mechanisms of resistance against β-lactams include expression of β-lactamases, alteration of membrane permeability and efflux, as well as target modification (8). Salmonella lacks chromosomally expressed β-lactamases, such as *ampC* but can gain mobile elements carrying extended spectrum β-lactamases (ESBLSs) (5, 9). Chromosomal mutations resulting in reduced membrane permeability or increased efflux are important determinants of extended-spectrum cephalosporin resistance and can also mediate cross-resistance to other classes of antibiotics (10, 11). Mutations that affect expression or confer structural changes to porins have been shown to have a direct effect on membrane permeability and hence antimicrobial resistance (12, 13). These changes are often seen in conjunction with increased production of multidrug-efflux pumps, able to extrude a wide range of toxic substrates from the cell to the environment (14–16).

Porin and efflux pump expression is carefully regulated and there are multiple mechanisms employed by the bacterial cell responsible for the fine tuning of membrane permeability. Expression of *acrAB* which has been studied as a prototype for efflux pumps is controlled by local repressor, AcrR and transcriptional regulators MarA, RamA and SoxS in Salmonella (17). Salmonella produces two general porins, OmpC and OmpF, and the balance of expression of the two is controlled by EnvZ-OmpR, a two-component system, in response to environmental stimuli such as osmolarity and pH changes (18).

In this work, we assessed how Salmonella evolves and adapts in response to cefotaxime exposure in two very different lifestyles: planktonic cultures and biofilms. We found that acquisition of mutations within a small region of *envZ* was the critical first step toward adaptation and development of resistance in all conditions. We show how these changes result in altered porin expression and reduced drug accumulation while also incurring a cost to other phenotypes important for fitness.

## RESULTS

### Cefotaxime exposure rapidly selects mutants with decreased susceptibility.

**FIG 1:**
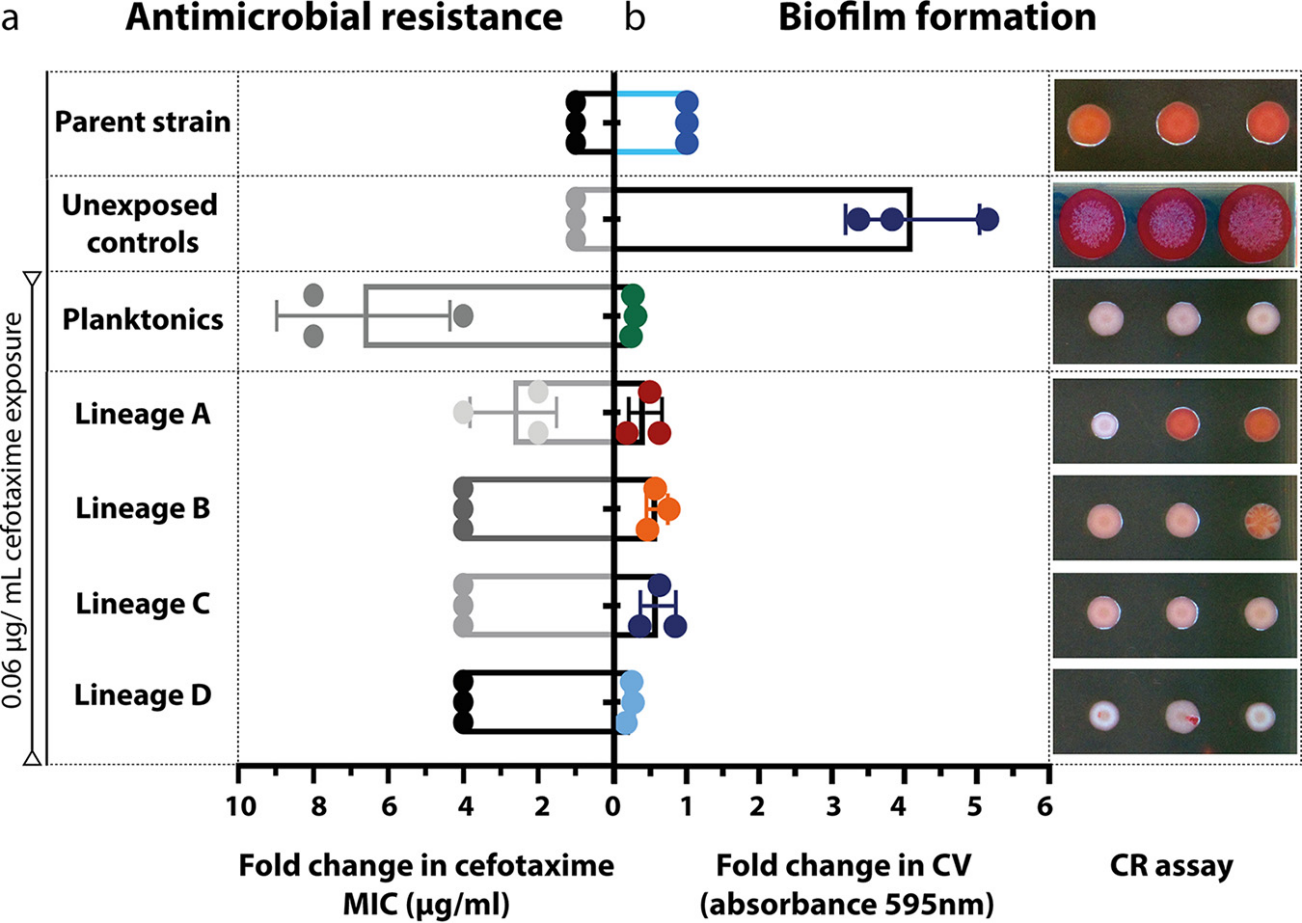
Cefotaxime sensitivity and biofilm formation of isolates. a, Bars show the average fold change in MIC of cefotaxime (μg/mL) for three, independent isolates from control, planktonic and biofilm-adapted lineages, compared to the WT. Dots show individual data points and error bars show standard deviation. b, Fold change in biofilm formation as measured by the crystal violet (CV) assay, compared to the WT. Dots show individual data points and error bars show standard deviation. Photographs show the morphology of three random isolates on LB-agar plates (with no salt), supplemented with congo red (CR).

Salmonella biofilms and planktonic cultures were exposed to 0.06 μg/mL of cefotaxime (1/2 MIC) over the course of 3 months being transferred into fresh medium supplemented with the antibiotic every 72 h. Biofilm and planktonic controls were also included where no drug was present (unexposed controls). At the end of the experiment, antimicrobial susceptibility was measured for 3 random colonies picked from each of four independent lineages, at three time points (designated early, middle, and late) across the experiment. The number of generations each lineage completed was estimated by multiplying the number of passages × log_2_ (the dilution factor), for biofilms the dilution factors were estimated by counting the average number of cells per bead obtained at the start and end of the experiments in control and drug-exposed lineages (from nine independent replicates). Control unexposed biofilms completed ~317 generations (with a range estimated from 282 to 330) while biofilms exposed to cefotaxime completed ~264 generations (range from 243 to 280), planktonic lineages completed ~170 generations (a lower value due to smaller dilution factor). The early, middle, and late time points for the exposed lineages correspond to approximately 31, 140, and 264 generations, respectively. Susceptibility was determined using the agar dilution method and biofilm formation was measured by the crystal violet (CV) (19) assay and by assessing colony morphology on agar plates supplemented with Congo red (CR) (Fig. 1). Unexposed control biofilm lineages did not show any change in antibiotic sensitivity (Fig. 1a) but adapted to the biofilm model and became significantly better at forming biofilms, as judged by a 4-fold increase in their biomass (Fig. 1b). No fixed mutations were identified in these strains. All independent lineages exposed to cefotaxime, developed resistance to the drug over time (Table 1). Planktonic cultures exhibited higher increases in the MIC of cefotaxime by the end of the experiment, when compared to biofilm lineages and reached the clinical breakpoint in some cases (2 μg/mL according to EUCAST) (Fig. 1a). Isolates exposed to cefotaxime (from both the biofilm and planktonic environments) formed weak biofilms as determined by biomass production by the CV assay and were characterized by a pale phenotype on CR plates (Fig. 1b). We confirmed these isolates were not contaminants by biochemical tests and genome sequencing which demonstrated they were isogenic to the parent.

**TABLE 1:** MIC (μg/mL) of cefotaxime against populations and strains isolated from biofilm and planktonic lineages recovered over time in the presence and absence of cefotaxime^*a*^

			Time point			
Condition	Drug^*b*^	Population/strain name	Start	Early	Middle	Late
Planktonic	−	P-control population	0.125	0.125	0.125	0.125
	−	P-ctrl- isolate 1	0.125	0.125	0.125	0.125
	−	P-ctrl- isolate 2	0.125	0.125	0.125	0.125
	−	P-ctrl- isolate 3	0.125	0.125	0.125	0.125
	+	Exposed population	0.125	0.5	**2**	**2**
	+	P-isolate 1	0.125	0.5	**2**	1
	+	P-isolate 2	0.125	0.5	**2**	1
	+	P-isolate 3	0.125	0.5	**2**	0.5
						
Biofilm	−	Control population	0.125	0.125	0.125	0.125
	−	Ctrl- isolate 1	0.125	0.125	0.125	0.125
	−	Ctrl- isolate 2	0.125	0.125	0.125	0.125
	−	Ctrl- isolate 3	0.125	0.125	0.125	0.125
	+	Population A	0.125	0.5	0.5	1
	+	A-isolate 1	0.125	0.5	0.5	0.5
	+	A-isolate2	0.125	0.5	0.5	0.25
	+	A-isolate3	0.125	0.5	0.5	0.25
	+	Population B	0.125	0.5	0.5	0.5
	+	B-isolate 1	0.125	0.5	0.5	0.5
	+	B-isolate 2	0.125	0.5	0.5	0.5
	+	B-isolate 3	0.125	0.5	0.5	0.5
	+	Population C	0.125	0.5	0.5	0.5
	+	C-isolate 1	0.125	0.5	0.5	0.5
	+	C-isolate 2	0.125	0.5	0.5	0.5
	+	C-isolate 3	0.125	0.5	0.5	0.5
	+	Population D	0.125	0.5	0.5	0.5
	+	D-isolate 1	0.125	0.5	0.5	0.5
	+	D-isolate 2	0.125	0.5	0.5	0.5
	+	D-isolate 3	0.125	0.5	0.5	0.5

a One representative of each control population is shown (results were identical in replicates). Values in bold reached the clinical breakpoint for resistance.

b “−” and “+” symbols indicate populations or isolates which were recovered after growth in the absence, or presence of cefotaxime, respectively.

### Multiple mutations within *envZ* underpin the evolution of cefotaxime resistance and compromised biofilm formation.

To investigate the genetic determinants responsible for the decreased susceptibility to cefotaxime and the loss of biofilm formation, we genome sequenced populations and individual isolates from passages 1, 9, and 17.

**FIG 2:**
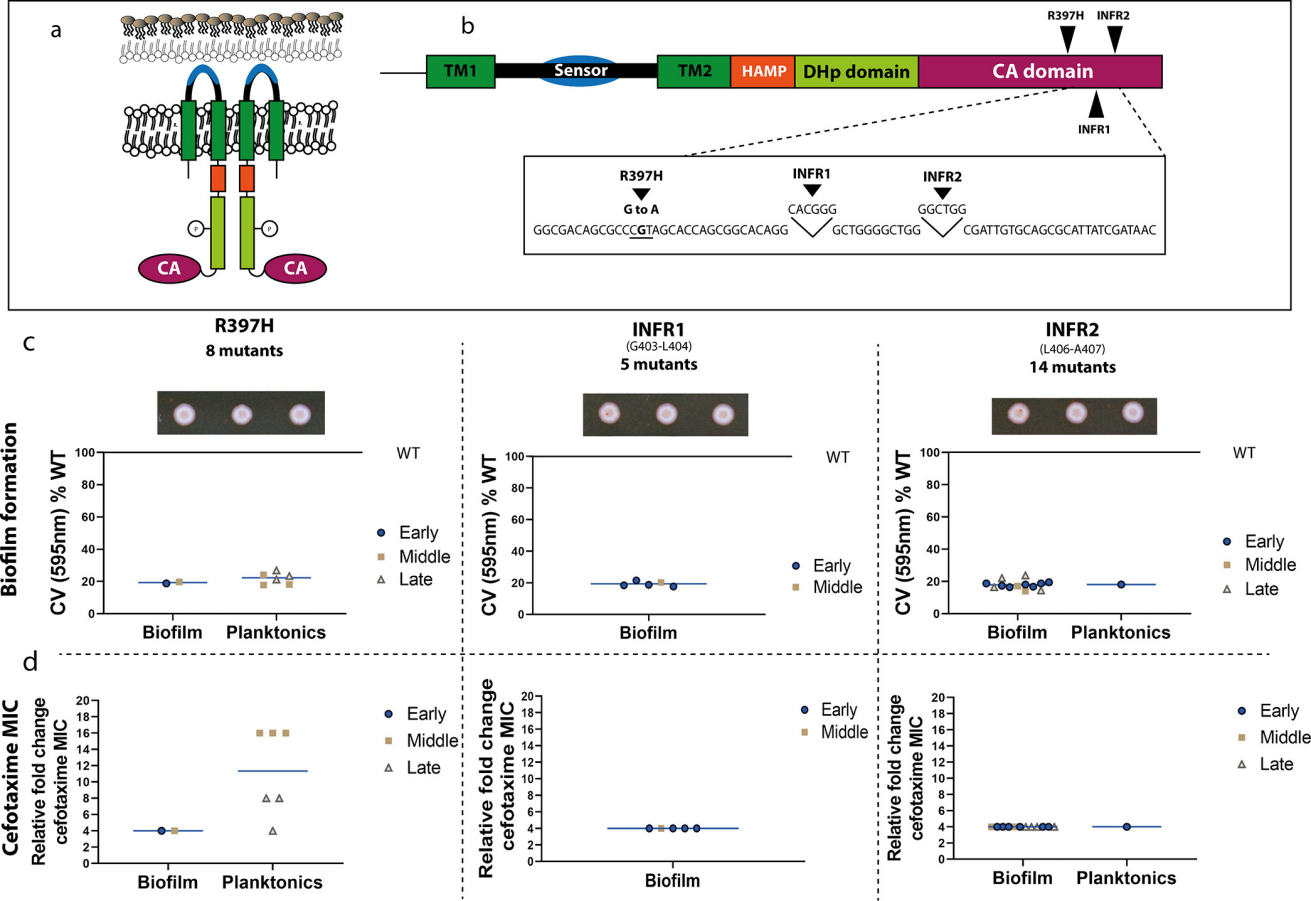
Domains of EnvZ with position and impact of changes. a, EnvZ is anchored to the inner membrane by two transmembrane regions (TM1-2, dark green). Between the two TM regions, there is a periplasmic sensor domain, which senses changes in osmolarity and other changes in the environment (black). Signals are transduced through the HAMP domain (orange) to the cytoplasmic domains of EnvZ. The DHp domain (light green) contains His-243, responsible for autophosphorylation (indicated with the P-sign). The C-terminal CA domain (purple) contains the catalytic/ATP-binding function. b, After exposure to cefotaxime three distinct envZ alleles emerged in multiple planktonic and biofilm lineages, all with changes around the ATPase active site of the protein: R397H, in frame insertion 1 (INFR1: CACGGG between G403 and L404), or in frame insertion 2 (INFR2: GGCTGG between L406-A407). Eight mutants were found to carry R397H substitution, 5 mutants INFR1 and 14 mutants carried INFR2. c, All mutants exhibited reduced biomass production compared to the WT, as measured by the CV assay. Isolates from the early time point are marked with blue dots, isolates from the middle time point are marked with yellow squares and isolates from late time points with gray triangles. Straight lines represent grand means. All isolates carrying mutations within envZ showed a distinct pale colony morphology on CR plates, three representative colonies of each genotype are shown. d, The MIC of cefotaxime was measured by the agar dilution method and fold changes relative to the WT were calculated. In most mutants, the MIC increased 4-fold, with the exemption of planktonic cultures carrying the R397H mutations that exhibited higher MICs over time (8 and 16-fold increase). These carried an additional substitution within AcrB (Q176K).

Sequencing of populations (3) and individual strains (3) recovered at each time point revealed that under cefotaxime exposure, there were an average of 7.3 SNPs per strain (rising from 4.7 to 9 over time) in biofilm lineages, and 4.6 SNPs per strain in planktonic conditions (rising from 3 to 7 over time). The rate of SNPS per strain per generation was the same between conditions (planktonic strains having completed fewer generations than biofilms). While mutations in various genes were found it was striking that changes within *envZ* were repeatedly seen in independent lineages. We did not deep sequence the populations and low abundance mutations within the populations may have been missed, however it seemed clear that mutation of *envZ* is the primary mechanism of cefotaxime resistance in these conditions. Therefore, to understand the role of *envZ* changes in cefotaxime resistance in more detail, we sequenced this locus from a wider number of individual isolates; we first sequenced 19 isolates from passages 1, 9, and 17. Among these, those with the pale-phenotype on Congo red plates that exhibited high drug tolerance all contained mutations within a small region of *envZ*. We then selected a further 22 isolates from different time points, based on their morphology on CR plates (pale colonies) and their resistance profile, and genotyped *envZ*. We found that of the 41 strains sequenced in total, 27 had mutations within *envZ*. All these changes clustered within a small region of the gene (Fig. 2a and b), with eight mutants carrying a mutation causing substitution R397H in EnvZ, five mutants had a conservative in frame 2-codon insertion of CACGGG between G403-L404 (referred to as INFR1), and 14 mutants carried a conservative in frame insertion of GGCTGG between amino acids L406-A407 (‘INFR2’). The R397H mutation was present in six planktonic and two biofilm isolates. INFR1 was only observed in biofilms and INFR2 was present in 13 biofilms and one planktonic culture. We also identified a single nucleotide substitution (resulting in S42R) in one biofilm isolate, exhibiting reduced biomass production (OD_595nm_: 0.101 ± 0.003) and increased tolerance to cefotaxime (MIC: 0.5). No mutations within *envZ* outside the relatively small hot spot region were identified.

The substitutions within EnvZ were associated with reduced biomass production as measured by the CV assay (10-20% of the WT’s biofilm capacity in most cases) and pale colony morphology on CR plates (Fig. 2c). The MIC of cefotaxime increased by fourfold in most mutants with the exemption of planktonic cultures carrying the R397H substitution where the cefotaxime MIC increased over time to eight (after nine passages) and then 16-fold (after 17 passages) (Fig. 2d). These planktonic mutants with higher MICs carried an additional mutation resulting in a change within the efflux pump AcrB (Q176K).

Mutations within EnvZ emerged in different time points throughout the experiment (Fig. 2), individual isolates were confirmed to be isogenic to the parent strain by genome sequencing. Substitutions within EnvZ emerged in multiple lineages in parallel but were unique to lineages exposed to cefotaxime and were not observed in any other selective pressures applied in previous studies from our group that used identical experimental setups (20).

### Cefotaxime resistance mutations are localized at the ATPase active site of EnvZ with a potential impact on the activity of the enzyme.

**FIG 3:**
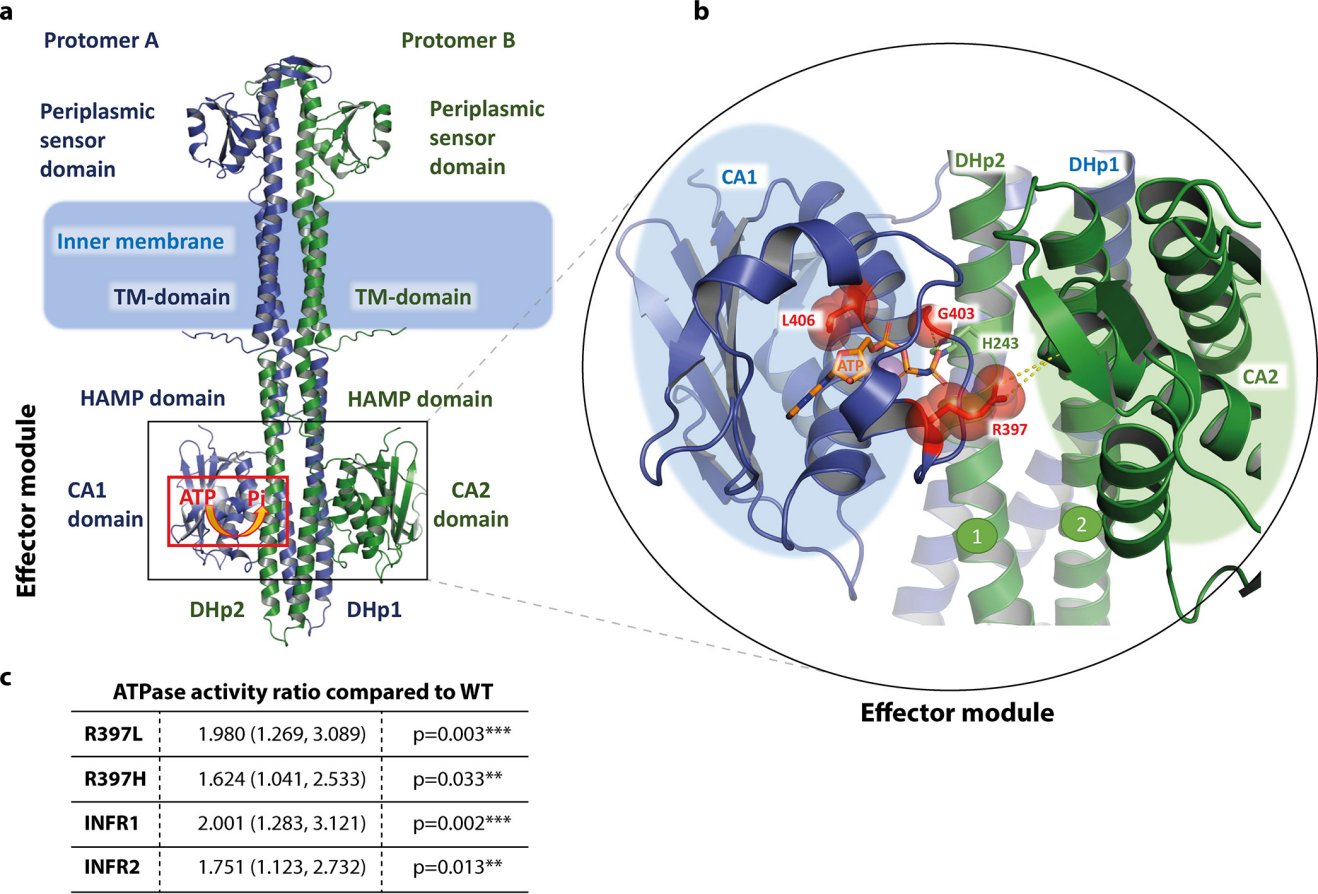
Structural analysis of changes within EnvZ and ATPase activities. a, *In silico* model of the EnvZ dimer showing the domains and their cellular locations. b, Close-up view of the mutated residues highlighting their possible role in ATP-coordination and catalysis, as well as CA-DHp and CA-CA-domain interactions. The side chains of R397, G403 and L406 residues (where changes were observed) are highlighted as sticks and spheres, and the location of ATP is shown. c, ATPase activity determination in EnvZ mutants as measured by the PK/LDH linked assay. Specific activity was calculated in nmoles of ATP hydrolyzed, per minutes, per mg of protein, in a range of ATP concentrations. Table shows the activity ratio for each mutant compared to the WT and associated *P* values for each. Data calculated from 7 biological replicates, each with three technical replicates per mutant.

To interpret the impact and downstream effects of the selected EnvZ mutations, we used *in silico* structure prediction (Fig. 3) to map their location within the EnvZ molecule, based on the recently released AlphaFold 2 models (21) and additional structural modeling as detailed in Materials and Methods.

EnvZ is a prototypical sensor histidine kinase, a membrane-spanning homodimer with the sensor domain spliced between two trans-membrane helices located in the periplasm. The cytoplasmic portion is composed of two structurally conserved cytoplasmic domains; the helical linker (HAMP) domain and the effector module (22, 23). The effector module itself is composed of a dimerization/histidine phosphorylation (DHp) domain and a C-terminal catalytic/ATP-binding (CA) domain, which are required for autophosphorylation in *trans* (24, 25) (Fig. 3a). Within the CA-domain, ATP-binding is provided by several conserved residue boxes, including the H-box (residues 243–248), N-box (residues 343–350), the ATP-lid (residues 385–397) and the G2-box (residues 401–405) (25). Phosphate from the ATP bound to the CA-domain is first transferred to a conserved histidine residue H243 on the DHp domain (*in-trans* autophosphorylation) and from there to an aspartate residue on the response regulator OmpR (phosphotransfer). *In silico* analysis showed that all three mutations identified localized to the CA-domain of the enzyme (effector module) (Fig. 2a and b, Fig. 3b). To assess their possible role in the mechanism of EnvZ action, we made local homology models based on the 4KP4.pdb (25) and 4CTI.pdb structures (26), which represent activated forms of the kinase. Crucially, we found that the mutations map into the conserved ATP-lid (R397H) and the G2-box of the CA-domain (INFR1 and INFR2), which play a critical role in ATP-coordination (25, 26). In addition, the sidechain of R367 reaches to the neighboring CA-domain of the second protomer (25), plausibly providing additional allosteric coordination of the autophosphorylation. The insertions INFR1 and INFR2, in addition to direct influence on the ATP-binding and kinase activity could possibly impact the packing of the CA-domain onto the DHp-helix 2 (25), potentially affecting the processivity of the enzyme.

Based on their location, we hypothesized that these mutations may have impacted the ATPase activity of the enzyme which could explain the downstream changes in phenotype (EnvZ activity being known to affect both porin (22, 27) and curli biosynthesis (28), which relate to drug accumulation and biofilm formation, respectively.

To do this, we expressed and purified the cytoplasmic soluble fraction of EnvZ, covering amino acids 180–450 (HAMP-domain and the effector module, composed of the DHp and CA-domains) by nickel affinity chromatography. Mutants R397H, INFR1, and INFR2 were reconstituted using molecular cloning techniques and were purified together with the WT version of the protein. We also purified mutant R397L, which has been previously described as positively impacting kinase activity of EnvZ (29).

We then tested the ATPase activity of all these protein variants using the pyruvate kinase/lactate dehydrogenase (PK/LDH) linked assay, which measures the hydrolysis of ATP through the oxidation of NADH via the pyruvate kinase (PK) and lactate dehydrogenase enzymes (LDH). Seven biological replicates were completed with freshly purified proteins in each run and 3 technical replicates included per condition to give a total of 21 replicates per protein variant. Although there was variation between the biological replicates, all the mutants exhibited higher average activity (60–100% increases) than the WT at ATP = 1 mM (Fig. 3c). While each mutant was different from WT, there was little difference in activity between mutants.

### Reconstitution of the R397H mutation confirms a role in drug tolerance with a tradeoff to biofilm formation.

To confirm the biological role of mutation at the ATPase site of *envZ* and to verify predicted impacts on porin and curli expression we selected the R397H substitution for further characterization by testing the phenotypes resulting from this substitution. This change emerged in both planktonic and biofilm lineages and was associated with highest MICs of cefotaxime.

**FIG 4:**
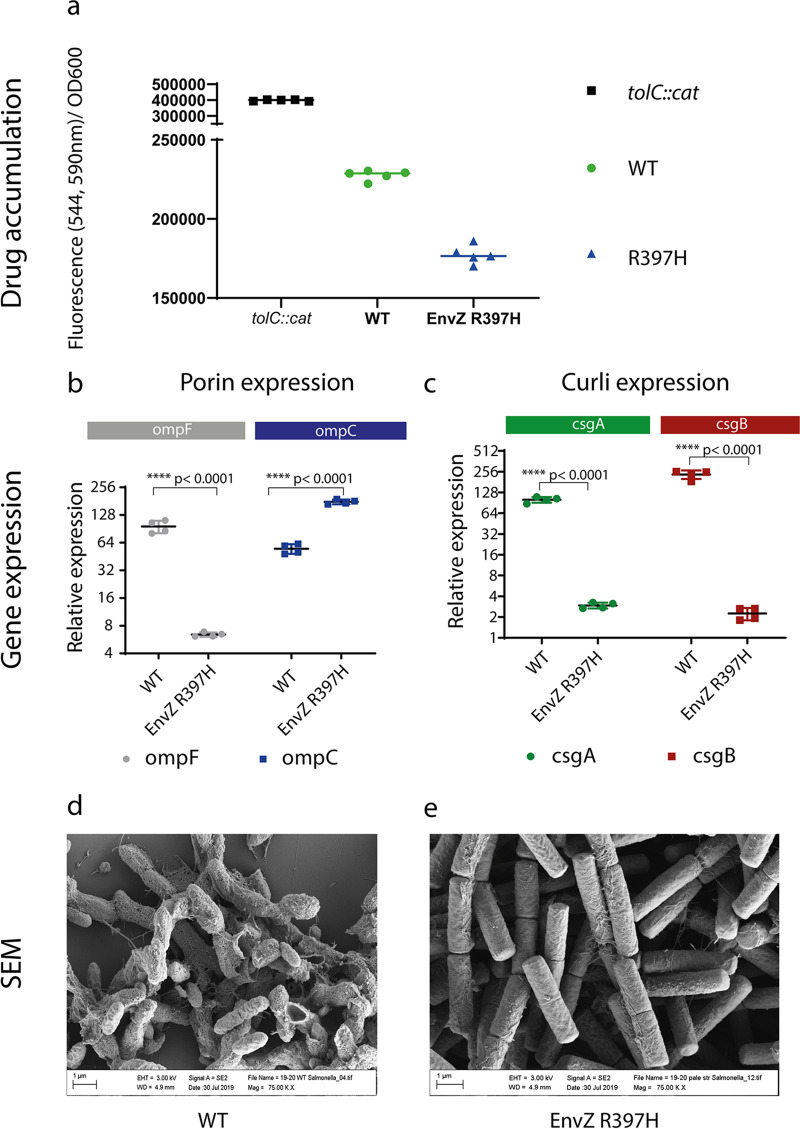
Phenotypic impact of EnvZ R397H substitution. a, Drug accumulation as measured by resazurin fluorescence. Lines indicate average values from 6 replicates, tolC mutant is an efflux deficient control. b, Relative expression of ompF and ompC measured by qRT-PCR from 48-h old biofilms (using gyrB as a reference) showing reduced expression in response to mutation of envZ. Error bars represent +/- one standard deviation. c, Expression of csgA and csgB was compromised in envZ mutants. Strains are named as in (b). Error bars represent +/- 1 standard deviation. d,e, Scanning Electron Microscopy (SEM) of 48-h biofilms grown on coverslips. Matrix formation is clearly observed in the WT strain (d) whereas in strains carrying the EnvZ R397H substitution (e), matrix formation is reduced.

We first measured drug accumulation of a strain carrying the identified substitution; EnvZ R397H. We used the WT-parent strain (14028S) as our reference and an isogenic *tol*C, efflux-deficient mutant as a control (*tol*C::*cat*). Membrane permeability was measured by monitoring resazurin intake by the cells. Resazurin is a nonfluorescent dye and efflux substrate which, when entering the cells, undergoes a redox reaction leading to color change. We observed that strain carrying the EnvZ substitution exhibited significantly lower drug accumulation compared to the WT (*P* < 0.0001) and the *tol*C mutant had increased drug accumulation, as expected (Fig. 4a).

Having confirmed that both mutations affect drug accumulation, we measured porin expression (*ompC* and *ompF*) by qRT-PCR (Fig. 4b), using *gyrB* expression as a reference. We found a significant reduction in *ompF* expression, which was correlated with a corresponding overexpression of *ompC* in the R397H mutant indicating that the balance in porin expression is altered in the mutant background. Such a change in the porin composition would be expected to confer decreased susceptibility to cefotaxime, indicating a K+P- state of EnvZ and is consistent with the observed phenotype.

To test whether biofilm formation was compromised due to reduced curli production, we measured expression of the main curli subunits, *csgA* and *csgB,* and found that expression of both in the *envZ* mutants was compromised (Fig. 4c). This explains the pale phenotype on CR and supports our hypothesis that selection of mutants with altered EnvZ function confers protection against cefotaxime, but at a cost to biofilm formation.

To further investigate the morphology of biofilms built by strains carrying the EnvZ mutation, we performed Scanning Electron Microscopy (SEM). We observed that in comparison to the WT strain, which exhibits a dense and “fibrous” matrix around each cell (Fig. 4d), cells carrying the EnvZ mutation were unable to form a normal matrix (Fig. 4e). This supports the q-RT PCR results showing curli expression in this mutant is compromised and this has a direct effect on the biofilm’s matrix production and colony morphology.

We further confirmed the specific phenotypic impacts of these two substitutions by creating isogenic mutants of the parent strain lacking *envZ* and complemented these with either wild-type or mutant alleles of *envZ* to determine the resulting impacts on phenotypes (Table 2). Deletion of *envZ* and complementation with the WT allele did not lead to any MIC changes for any of the antibiotics tested. However, complementation with the EnvZ R397H allele led to a 4-fold increase in MICs of cefotaxime, chloramphenicol, and tetracycline.

**TABLE 2:** Susceptibility to a panel of antibiotics of defined mutants with specific gene deletions and complementations to validate impact of proposed mechanisms of resistance

	MIC (μg/mL)						
Strain	Azi^*a*^	Cef	Chl	Cip	Kan	Nal	Tet
WT	4	0.125	4	0.03	4	2	0.5
EnvZ R397H	4	0.5	16	0.06	2	4	1
Δ*env*Z	4	0.125	8	0.03	4	2	0.5
Δ*env*Z :: envZ	4	0.125	ND	0.03	ND	2	0.5
Δ*env*Z :: envZ R397H	8	0.5	16	0.06	ND	4	1

a Azi, azithromycin; Cef, cefotaxime; Chl, chloramphenicol; Cip, ciprofloxacin; Kan, kanamycin; Nal, nalidixic acid; Tet, tetracycline. ND, not determined (due to presence of a corresponding resistance cassette).

Together, these results confirm that the R397H mutation results in altered activity of EnvZ and subsequent repression of OmpF production (the major influx channel for cefotaxime), however the mutation also resulted in a loss of biofilm matrix production.

### Cefotaxime resistant mutants within biofilms represent a subpopulation.

**FIG 5:**
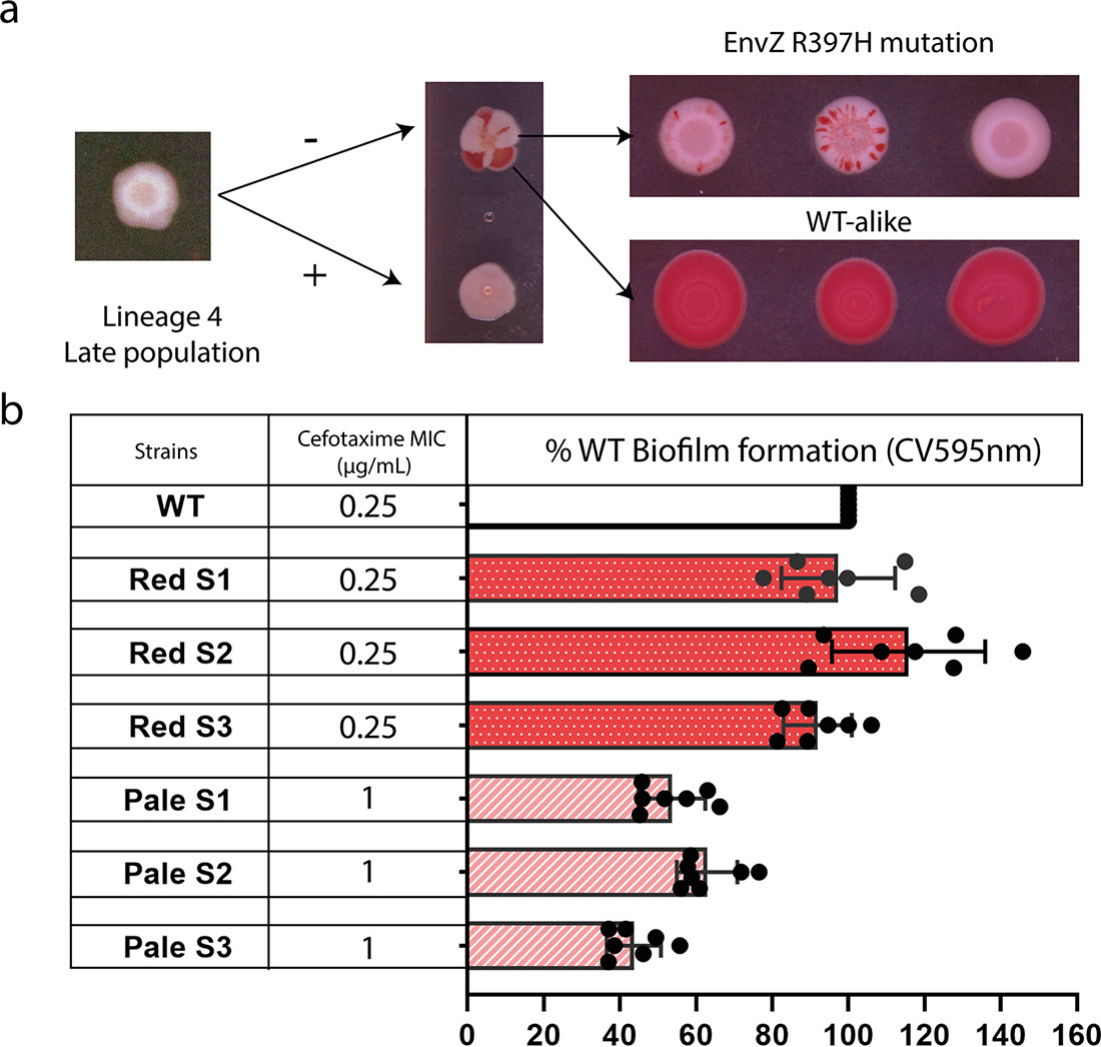
Heterogeneity and phenotypes of cefotaxime-exposed populations. a, A pale population isolated from the last passage of the evolution experiment (exposed to cefotaxime) was selected. When passaged in the absence of cefotaxime, a red (WT) subpopulation becomes more prevalent, whereas in the presence of the drug, the pale population remains fixed. Three single isolates representing each subpopulation were spotted onto CR plates with no selection. The red colonies remained phenotypically uniform; the pale colonies however showed diversity with emergence of red cells within the resulting colonies. b, The MIC of cefotaxime for the red colonies was identical to the WT (0.25 μg/mL), whereas the pales exhibited a fourfold increase in their MIC (1 μg/mL). Biofilm formation was measured by the CV assay (absorbance at 595 nm) for both the red and the pale single colonies, with the pales exhibiting compromised biofilm ability compared to the reds and the WT. Seven technical replicates were included for each single colony. Whole genome sequencing showed all three pale colonies carry the EnvZ R397H substitution, whereas the red ones were genetically identical to the WT.

While cefotaxime readily selected for resistant mutants, these were poor biofilm formers and produced a distinct pale phenotype on CR plates. Similar mutations were recovered from populations exposed to cefotaxime in both planktonic and biofilm conditions. This suggested that while the mutants were able to survive drug exposure they may have significant fitness defects in biofilm conditions. Analysis of populations revealed heterogeneity was present with two morphology types of present; ‘pale’ and ‘red’, named according to the morphology of colonies on Congo red plates. This suggests that resistant mutants (the pale type) emerge but do not sweep to dominance within a population within the timescale of the experiments completed here. To study the phenotypes of each population further, we selected an end stage biofilm population known to contain both WT cells and pale mutants (with the R397H mutation) to seed experiments to determine the prevalence of pale and red mutants in populations grown under different conditions. We used this initial population to inoculate replicate populations in broth to allow re-growth in the presence and absence of cefotaxime and after 24 h spotted cells from each population on CR plates (Fig. 5a). We observed that, in the presence of cefotaxime populations appeared uniformly pale suggesting the pale subpopulation had a fitness benefit over the red. Populations grown in the absence of cefotaxime demonstrated a mix of the pale and red subpopulations. We picked three single colonies of each morphology for further phenotyping and characterization (Fig. 5a). We observed that all red colonies had the same MIC for cefotaxime as the WT, while all pale colonies had an 4-fold increase in cefotaxime MIC. Red colonies, also formed biofilms comparable to the WT, whereas the pales formed weaker biofilms as measured by the CV assay (Fig. 5b). We whole genome sequenced all six isolates (three pales and three reds) which confirmed that all red colonies were genetically identical to the WT, whereas all pale colonies carried the EnvZ R397H mutation.

### Drug exposure encourages expansion of the resistant subpopulation, which is out-competed by the WT strain, in the absence of any drug selection.

To further characterize potential fitness advantages and disadvantages of the pale subpopulation within a population under different conditions, we performed an accelerated evolution experiment of an R397H mutant in competition against the WT (14028S with *lacZ* incorporated allowing blue/white selection). The WT was mixed in a 1:1 ratio with the pale mutant and populations then passaged in the presence and absence of cefotaxime for ten 24-h passages, in both planktonic and biofilm environments.

**FIG 6:**
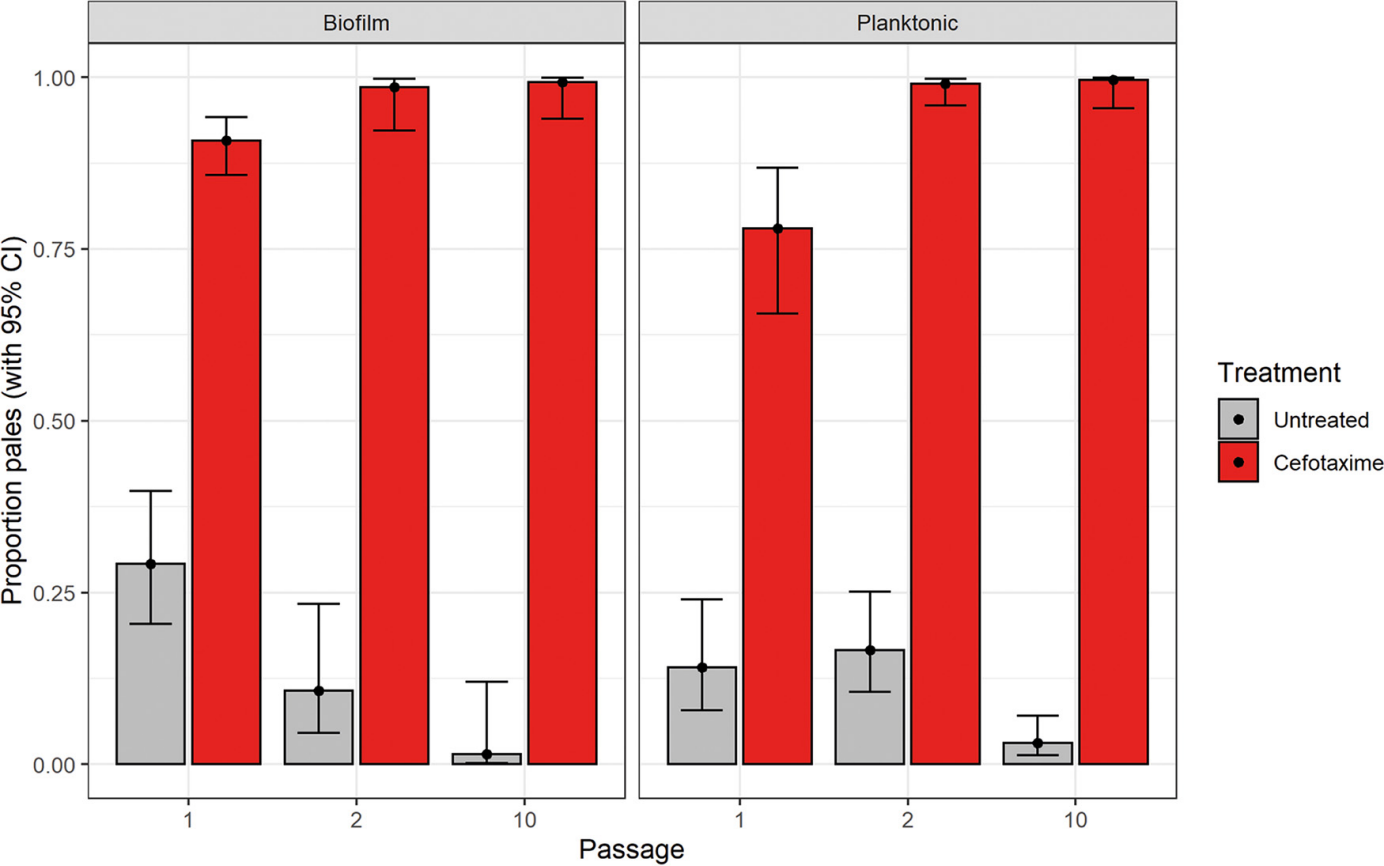
Accelerated evolution experiment to characterize competitive fitness of the envZ mutant against the WT genotype. A tagged WT (14028S::lacZ) was mixed, in a 1:1 ratio, with a pale isolate (carrying the EnvZ R397H substitution) and passaged overnight before the resulting culture was used to inoculate fresh media. This was repeated for ten 24-h passages, six experiments were replicated in planktonic conditions and six in biofilm conditions; in each condition half the lineages were exposed to 0.032 μg/mL cefotaxime and half had no drug present. Bars show the proportion of pale relative to WT strains (with a 95% confidence interval,) throughout the course of the experiment in each condition. Results show cefotaxime strongly selects for a high prevalence of pale colonies in both planktonic and biofilm environments whereas, in the absence of drug the WT dominates.

In both biofilm and planktonic conditions in the presence of the drug, the pale subpopulation had a clear advantage over the WT and became the prevalent population within the community by the end of the experiments. Conversely, in the absence of any selection pressure, the WT had a clear fitness advantage over the pale subpopulation and the same responses were observed in both planktonic and biofilm environments (Fig. 6a and b). No population completely lost either genotype under any condition.

## DISCUSSION

Understanding evolution of resistance is crucial to prevent emergence of multidrug resistant strains and can inform future strategies toward clinical interventions. One of the most well-studied mechanisms for resistance against β-lactams is the acquirement of mobile elements carrying b-lactamase enzymes. In recent years multiple plasmids carrying enzymes degrading β-lactams have been identified in Salmonella (4, 9, 30, 31). However, chromosomal resistance mechanisms are also crucial to development of resistance with control of drug accumulation (by efflux or porin control) being an underpinning mechanism without which many other mechanisms of resistance become inactive (32).

The aim of the present study was to determine how Salmonella can evolve resistance against β-lactams, focusing on chromosomal alterations and whether growth in a biofilm favored different pathways to growth in planktonic conditions.

We identified various mutations in lineages exposed to cefotaxime with multiple separate changes seen within a small hot spot within *envZ* from independent lineages and in different conditions and time points. While our experimental design could not rule out the presence of other important mutations in the populations at low frequency, the high prevalence of changes with *envZ* and the fact that all *envZ* mutants identified had increased tolerance to cefotaxime strongly suggests their role as a primary mechanism of cefotaxime resistance.

The mutations identified were the R397H single amino-acid substitution, the in-frame insertion INFR1-CACGGG (between G403-L404), and the in-frame duplication INFR2-GGCTGG (between L406-A407). Structural analysis of the mutations revealed that they all map within the C-terminal catalytic site of EnvZ, laying within a flexible loop near the residues implicated in nucleotide coordination at the ATPase active site. In particular, residues 392–393 and 402–406 form the ATP-binding site (24, 26, 33).

EnvZ is part of a two-component system with OmpR, which controls the differential expression of *ompF* and *ompC* in response to changes in osmolarity in the environment (22, 23). EnvZ possesses both kinase and phosphatase activity, which are facilitated by ATP hydrolysis. It auto-phosphorylates itself at His-243 and then transfers the phosphate to OmpR to activate it. The protein can exist in two active but opposed states, the kinase-dominant state (K+P-), which leads to increased levels of OmpR-P in the cell and the phosphatase-dominant state (K-*P*+) (34). It is thought that low levels of OmpR-P activate expression of *ompF*, whereas *ompC* expression (and *ompF* repression) occurs at high levels of OmpR-P (35). Another study suggested that the kinase activity of the enzyme remains constant, whereas changes in the phosphatase activity of the enzyme may be responsible for alterations in OmpR-P levels (36).

Based on the above considerations, we hypothesized that the emergence of mutations within this region would have a direct effect on the ATPase activity, impacting activation of OmpR by EnvZ and resulting in altered membrane permeability and consequent decreased susceptibility to cefotaxime. Due to the presence of transmembrane regions within EnvZ, it is not feasible to purify the full-length enzyme in physiologically relevant form. We therefore purified and tested the cytoplasmic region of EnvZ, based on previous work (29), for its ATPase activity and compared it to purified alleles carrying the identified substitutions. All the mutants tested here showed a modest, but consistent increase in ability to turn over ATP (Fig. 3c) compared to the WT, including the mutant with the R397L substitution, consistent with it being previously reported to result in an increased population of phosphorylated OmpR (29).

Additional characterization of the R397H mutant, showed this mutant exhibited reduced *ompF* and increased *ompC* expression, while having a direct impact on membrane permeability, as tested by the resazurin accumulation assay. These results provide additional evidence that changes we see in EnvZ result in high levels of OmpR-P in the cells, consequent lowered OmpF levels and ultimately reduced permeability in the cell.

While we observed an increase in ATP turnover, and detected increased OmpC levels and reduced OmpF expression, which is consistent with an interpretation of the mutation causing increased ATPase activity, and correspondingly a K+P- state of EnvZ, it is not possible to say with certainty from our data whether the EnvZ variants we see act by changing their kinase or phosphatase activity, and it is not feasible to study the full-length protein in physiological conditions. However, th*e in-silico* analysis of the possible impact of these substitutions on the structure of EnvZ suggests some mechanistic possibilities. Previously altered activity of the R397L mutant has been attributed to potentially reduced phosphatase activity due to the observed increase in OmpR-P levels on the R397L mutant (29). While none of the mutations identified here impact the phosphoacceptor H243-site itself, some regions within close proximity of the mutations identified have been shown to affect kinase activity of the enzyme, while not affecting phosphatase activity (e.g., F390L) (37). R392 has also been directly implicated in the ATP-binding (26). The location of the mutations within the CA-domain as discussed above might explain their impact on the EnvZ activity (Fig. 3). A simplistic explanation of their effect could be that they directly affect the ATPase activity of EnvZ due to their involvement in ATP coordination, with L406 being located right next to G405 which coordinates the gamma-phosphate of ATP, while G403 is directly involved in ATP-coordination itself as could be seen in the available crystal structure (4KP4.pdb) (25). However, in addition to direct influence on the ATP-binding and kinase activity the insertions INFR1 and INFR2 could impact the packing of the CA-domain onto the DHp-helix 2, possibly affecting the processivity of the enzyme. Indeed, it has been previously proposed that altered recognition of the catalytic domain by DHp, rather than a shift in position of the phospho-accepting histidine, forms the basis for regulation of kinase activity (38), and it is not implausible that INFR1 and INFR2 may impact the CA-DHp packing. Furthermore, subsequent studies have proposed a sequential flip-flop autokinase mechanism for EnvZ (26), where the DHp-bundle alternately links upon docking of a CA-domain on either side. There, the CA-domains are suggested not to interact with the DHp domain in the region of the bundle-tip, but to stay associated with it at the height of the so-called “DRT-motif,” above the catalytic histidine H243. As the response regulator OmpR is suggested to bind at the DHp-bundle tip below the H243, it has been suggested that the kinase could be active when the interaction with the OmpR is already established (26). Similarly, the R367 sidechain reaches to the neighboring CA-domain of the second protomer, plausibly providing additional allosteric coordination of the autophosphorylation.

The mutants isolated in our study could cause several structural impacts on EnvZ, however it remains to be determined whether the effect observed is fully due to increase of the kinase activity, suppression of the phosphatase activity or affects the docking and stability of its association with the response regulator.

Interestingly, the mutations characterized above were not shared by all the cells within a population. In fact, we observed that these were present in a subpopulation, which never became fully dominant under the conditions tested although the experiments were limited in the number of generations tested. Populations exposed to cefotaxime selected for pale, *envZ* mutants which expanded in the presence of the drug although the populations also maintained red (WT) strains although these only became detectable from drug-exposed lineages after subsequent passage in drug free media. This maintenance of both genotypes may allow resilience for the population as the pales are significantly compromised in biofilm ability. Beyond the phenotypes studied in detail here, we also identified changes in morphology of EnvZ mutants by electron microscopy suggesting other pleiotropic effects result from selection of these mutations.

Analysis of the Enterobase database identified the presence of the INF2 substitution in an *S.* Heidelberg isolated in 2019 in a chicken in the USA demonstrating that this genotype is seen in real world conditions. The other changes identified were not seen, future work can assess how common these genotypes are in isolates and whether there is a differential abundance related to individual fitness costs. Studying the dynamics of how different genotypes can compete or co-operate in different conditions is important in understanding how antibiotic resistance emerges and in predicting which mutations will be successful in different conditions.

## MATERIALS AND METHODS

### Biofilm adaptation and evolution model.

The biofilm bead model used was as previously described (39). Briefly, Salmonella biofilms were grown on glass beads (6 mm soda lime, Sigma, Z265950-1EA), for 72 h in 5 mL lysogeny broth (LB) with no salt at 30°C and were serially transferred for 17 passages. For stressed lineages experiments included 0.06 μg/mL cefotaxime. Eight lineages were included: two unexposed biofilm control lineages, two exposed planktonic lineages and four independent cefotaxime-exposed biofilm lineages. The beads were washed in PBS between passages and a 1:1000 inoculum was used for the planktonic lineages. Biofilm populations were isolated from the beads by vortexing and were stored in 20% glycerol for subsequent phenotyping. Three single colonies were isolated from each biofilm population and one of the control lineages, these were also stored in deep-well-plates and were phenotyped by replication of the plate onto appropriate media.

### CV assay.

Strains were grown O/N and diluted to an OD of 0.01, in microtiter plates at a final volume of 200 μL. They were incubated for 48 h at 30°C, covered with gas-permeable seals. The wells were emptied and rinsed with sterile water and were then strained with 0.1% CV. They were incubated with the dye for 15 min before they were rinsed again with water. The dye bound to the cells was dissolved in 200 μL of 70% ethanol and the absorbance was measured at 595 nm in a plate reader (FLUOStar Omega, BMG Labtech).

### CR assay.

Cells were grown in LB overnight, diluted to a final OD of 0.01 and 2 μL spots inoculated onto 1% agar plates with LB with no salt, supplemented with 40 μg/mL CR dye. They were incubated for 48 h at 30°C before capturing the colony morphology by photography.

### Antimicrobial susceptibility testing.

Minimum inhibition concentrations of antibiotics were determined by the broth microdilution method (40) and the agar dilution method (41), following the EUCAST guidelines, using Mueller-Hinton broth or agar.

### Drug accumulation assays.

To detect differences in cellular permeability to drugs between mutants, the resazurin accumulation assay was used. The strains of interest were grown to exponential phase, using a 1:100 inoculum from overnight cultures. The cells were washed and resuspended in PBS normalizing for cell density, and they were mixed with resazurin to a final volume of 100 μL (to give 10 μg/mL) in round-bottom microtiter plates. Fluorescence was measured in the Omega FLUOstar plate reader at excitation 544 nm and emission of 590 nm. Five replicates were included per strain and resazurin-only reactions were used as controls. The assay was repeated at least twice with reproducible results observed each time.

### Preparation of RNA samples for q-RT PCR.

RNA from biofilms was isolated using the SV Total RNA Isolation System kit (Promega). RNA was extracted from strains grown O/N at 37°C in LB and then spotted on LB-NaCl agar plates and incubated for 48 h at 30°C. Cells from each spot were then resuspended in 100 μL TE containing 50 mg/mL lysozyme and were homogenized by vortexing. Seventy-five microliter of RNA Lysis Buffer (Promega kit), followed by 350 μL RNA Dilution Buffer (Promega kit) were added to the cell suspensions, which were then mixed by inversion. Samples were incubated at 70°C for 3 min and centrifuged at 13,000 g for 10 min. The supernatant was mixed with 200 μL 95% ethanol and was then loaded on to the spin columns provided by the kit. The columns were washed with 600 μL RNA Wash Solution. DNase mix was prepared following the Promega kit protocol and 50 μL were directly added on the column membrane. After a 30 min incubation, 200 μL DNase Stop Solution was added and samples were centrifuged for 30 s. Columns were washed with 600 μL RNA Wash Solution followed by 250 μL RNA Wash Solution, and then centrifuged again for 1 min to dry. RNA was eluted using 100 μL of nuclease-free water. RNA quantification was performed using the Qubit RNA High Sensitivity assay kit (Q32852).

### q-RT PCR.

To determine expression levels of *ompC/F*, *csgA/B* and *ramA,* we used q-RT PCR using the Luna Universal One-Step RT-qPCR Kit from NEB (E3005), and an Applied Biosystems 7500 real-time PCR system. The primers used for the q-RT PCR are listed in Table S3. Efficiency of the primers was calculated by generation of calibration curves for each primer pair on serially diluted DNA samples. The R^2^ of the calibration curves calibrated was ≥0.98 for all the primer pairs used in this study.

RNA at a final amount of 50–100 ng was added to 10 μL final volume PCRs, mixed with 400 nM each primer. The cycle parameters were as follows: 10 min at 55°C (reverse transcription step), 1-min denaturation at 95°C and 40 cycles of 10 s at 95°C and 1 min at 60°C.

For each sample, two technical replicates from two biological replicates each were included (four in total) per reaction. Controls with no reverse transcriptase were also included for each RNA sample to eliminate DNA contamination.

To calculate expression levels, expression fold change was calculated using *gyrB* expression as a reference. The relative expression was determined by calculating the logarithmic base 2 of the difference between *gyrB* gene expression and target gene expression per sample.

### SEM and sample preparation.

Biofilms of the parent strain (14028S) and of an isolate carrying the EnvZ R397H substitution, were grown on glass coverslips (agar scientific, AGL46R13-2) at 30°C for 48 h in LB with no salt. The cells on the slide were fixed in 2.5% glutaraldehyde in 0.1M PIPES at room temperature for approximately 3 h and then placed in the fridge overnight. The fixative was removed and replaced with 0.1M PIPES buffer (3× washes). After the buffer washes, the slides were taken through an ethanol series, starting at 30% then 50% then 70%, 80%, 90%, 100% and then 2× dry 100% ethanol. The glass slides were snapped in half (or thereabouts) using tweezers and a large sized piece of each that was supporting cells was transferred into a critical point drying (CPD) capsule. The cells were critical point dried in a Leica CPD300 with liquid CO2 as the transition fluid. The glass slide pieces, supporting the salmonella cells, were mounted on aluminum SEM stubs (Agar Scientific, Stansted, UK) using sticky carbon tabs (Agar Scientific, Stansted, UK). To ensure the slide pieces were cell-side-up, the pieces were examined under a WILD stereomicroscope and a corner of the cell layer gently scratched with tweezer tips to determine which side of the glass they were on. The stubs were placed a Leica ACE 600 sputter-coater and gold coated for 50 sec. The cells were observed and imaged in a Zeiss Supra 55 FEG SEM, operating at 3 kV.

### Molecular modeling and antibiotic docking.

The assembled model of the full length EnvZ presented in Figure 3a and b is based on the recently released AlphaFold 2 predictions (21) (PMID: 34265844), with local validation of the periplasmic domain based on its experimental structure in isolation (5XGA.pdb) (42) and docking orientation based on the dimeric structures of the periplasmic domains of KinD (4JGP.pdb) (43) and PhoQ (3BQ8.pdb) (44). The HAMP helical bundle was corroborated using the experimental structures (2LFS.pdb) (38). For the analysis of the effect of the mutations within the CA-domain of EnvZ, we used the experimental structure of the *E. coli* EnvZ fused to the catalytic part of Archaeoglobus fulgidus Af1503 HAMP-domain (4CTI.pdb) (26), which represents the activated kinase form to create a homology model using I-TASSER (45), while the ATP-bound form of the EnvZ shown in Fig. 3, was modeled based on the chimeric EnvZ-HK853 bound to the AMPPNP (4KP4.pdb) (25). All visualizations were performed with PyMol (The PyMOL Molecular Graphics System, Version 1.7, Schrödinger, LLC).

### Strains and genetic manipulations.

Escherichia coli DH10b was used as a host for all cloning procedures. Transformations of E. coli were carried out by heat shock of chemically competent E. coli cells. Transformation of Salmonella was carried out by electroporation. Salmonella electrocompetent cells were prepared as follows: Salmonella cells were grown to early exponential phase (OD_600nm_ 0.2–0.3) in 50 mL of 2× YT, using a 1:100 inoculum from an overnight culture. The cells were centrifuged and washed once with filter sterilized ice-cold water. They were left to incubate on ice for 1 h before they were pelleted at 3,000 g for 15 min. The cell pellet was resuspended in 1 mL of 10% filter-sterilized glycerol and 100 μL were used per transformation.

To create gene deletion mutants, we used the λ-red-based, gene doctoring technique previously described in (46). For each deletion, two homologous regions upstream and downstream of the genes of interest were amplified by PCR and were cloned in MCS1 and MCS2 of the pDOC-K vector. The homologous regions were 300–400 bp in length and were designed to include the first and last 10 codons of the gene to be deleted, to avoid any pleiotropic effects after deletion. For the *envZ* deletion, the upstream homologous region was cloned EcoRI/BamHI in MCS1 and the downstream one as XhoI/SpeI in MCS2.

For complementation of mutated genes, chromosomal integrations were created to insert wild-type copies or mutated versions of genes of interest. This used a modification of the gene doctoring system described above. pDOC-K was modified to be used to deliver chromosomal gene integrations to the intragenic region downstream of *glms*. The vector used for that was pDOC-K -glms as described in (47). Wild-type *envZ* and *envZ-*R397H alleles were cloned XhoI/HindIII in pDOC-K/glms under the control of a constitutive plac promoter.

For induction of chromosomal integrations either for deletion or complementation of a gene, the strain to be modified was transformed with the pDOC-K vector variant and the pACBSCE helper plasmid carrying the λ-red genes. A single colony carrying both plasmids was grown in 500 μL of LB, at 37°C for 4 h. The cells were pelleted and washed three times in filter sterilized LB. They were then resuspended in 500 μL of 0.1× LB supplemented with 0.3% arabinose and incubated at 37°C for 2–3 h for induction. 100 μL were plated on LB plates supplemented with 25 μg/mL kanamycin and 5% sucrose. The plates were incubated overnight at 37°C. Single colonies were checked for chromosomal alterations using colony PCR with primers annealing outside of the region to be modified. The plasmids were removed by subculturing the positive clones on kanamycin-supplemented plates and testing them for chloramphenicol and ampicillin sensitivity until the plasmids were completely removed.

For double deletions and/or complementations, the kanamycin cassette, introduced by the first chromosomal modification, was removed using the FLP sites flanking the cassette. The strains were transformed by electroporation with the pCP20 vector, carrying the genes for flippase activity, and recovered on LB agar plates supplemented with 50 μg/mL ampicillin at 30°C. The kanamycin cassette removal was confirmed by colony PCR and the positive clones were subcultured on LB agar at 37–42°C. Removal of the plasmid was confirmed by testing the colonies’ sensitivity to ampicillin.

### Accelerated evolution experiment.

An accelerated evolution experiment was used to assess stability of mutants with the EnvZ R397H substitution against the parent strain tagged with lacI/Z (*14028S::lacZ*) in planktonic and biofilm conditions. The two strains were grown O/N in LB with no salt and their ODs normalized to 0.5 before being used to inoculate populations 1:1. The experiment was performed on three planktonic cultures with no drug and three planktonic cultures with 0.032 μg/mL of cefotaxime. Passages were performed with a 1:1,000 dilution from the previous time point. For the biofilm environment, glass beads were inoculated with the initial mix of strains and one colonized bead was used to inoculate the next tube with sterile beads for the subsequent passages. Beads were washed with PBS between passages to avoid carry over of planktonic growth. Passages took place every 24 h and the experiment lasted for 10 passages in total. Cells from each condition were isolated at the end of each passage by vortexing. CFU for each strain were determined by serial dilution on LB plates supplemented with 40 μg/mL X-gal and 1 mM IPTG, to distinguish between the WT and the mutant strains.

### Statistical analysis.

Proportions of pale mutants are estimated from the accelerated evolution experiment by modeling the number of CFU observed using a generalized linear mixed model. A log-link was used with the number of dilutions as an offset variable. The effects of passage number, environment, cell type (pale or WT) and treatment were entered as fixed effects along with each of their 2- and 3-way interactions. A random effect corresponding to technical replicate was entered to account for over-dispersion. Models were estimated with the lme4 package version 1.1–21 using R statistical software version 3.6.1.

### Data availability.

Whole genome sequencing data that support the findings of this study have been deposited in the Sequence Read Archive (SRA) identifier SRP189875 with the project number PRJNA529870 (accession numbers: SAMN11288384, SAMN11288382, SAMN11288381, SAMN11288380, SAMN11288379, SAMN11288378, SAMN11288370, SAMN11288368, SAMN11288366, SAMN11288361).
